# Dosage, effectiveness, and safety of sertraline treatment for posttraumatic stress disorder in a Japanese clinical setting: a retrospective study

**DOI:** 10.1186/s12888-016-1138-5

**Published:** 2016-12-07

**Authors:** Toshiko Kamo, Masaharu Maeda, Misari Oe, Hiroshi Kato, Jun Shigemura, Kazuhiko Kuribayashi, Yuko Hoshino

**Affiliations:** 1 Institute of Women’s Health, Tokyo Women’s Medical University, Shinjuku, Tokyo, Japan; 2 Department of Disaster Psychiatry, Fukushima Medical University, Fukushima, Fukushima, Japan; 3 Department of Neuropsychiatry, Kurume University School of Medicine, Kurume, Fukuoka Japan; 4 Hyogo Institute for Traumatic Stress, Kobe, Hyogo Japan; 5 Department of Psychiatry, National Defense Medical College, Tokorozawa, Saitama Japan; 6 Clinical Statistics, Pfizer Japan Inc., Shibuya, Tokyo, Japan; 7 Clinical Research, Pfizer Japan Inc., Shibuya, Tokyo, Japan

**Keywords:** Posttraumatic stress disorder (PTSD), Sertraline, Selective serotonin reuptake inhibitors (SSRI), Antidepressant, Pharmacotherapy, Chart review, Japan

## Abstract

**Background:**

Many of the posttraumatic stress disorder (PTSD) treatment guidelines recognize the use of selective serotonin reuptake inhibitors as first-line pharmacological treatment. In Japan, there were no published studies investigating the effectiveness and safety of sertraline for PTSD in a clinical setting.

**Methods:**

We conducted a retrospective medical chart review of the dosage, effectiveness, and safety of sertraline for the PTSD treatment in Japan. Data were collected from medical charts of patients of PTSD, caused by various types of trauma, who were treated with sertraline between July 2006 and October 2012 during their regular clinical practice. To evaluate the effectiveness, the investigators retrospectively assessed the severity and improvement of the symptoms using the Clinical Global Impressions − Severity and the Clinical Global Impressions − Improvement.

**Results:**

The study population was 122 Japanese patients aged ≥18 years with a diagnosis of PTSD who were treated with sertraline (median duration, 10.6 months). Doses ranged from 12.5 to 150 mg/day, mostly 25 and 50 mg/day. The median duration of observation was 10.8 months. Out of those, 50% of patients were regarded as responders by using the Clinical Global Impressions - Improvement at the end of sertraline treatment or the last observation. Two-thirds (65.6%) of patients improved in the severity of PTSD, as assessed by Clinical Global Impressions - Severity, whereas 32.8% showed no change, and 1.6% worsened. Subgroups analyses and logistic regression analyses suggested that the type of traumatic events was the factor with the highest influence on the response rate. The adverse events in this chart review were consistent with the known safety profile of sertraline. There were no reports of serious or severe adverse events considered to be related to sertraline.

**Conclusions:**

Our study suggested the effectiveness of sertraline for the treatment of PTSD in a Japanese clinical setting, and the obtained safety profile was consistent with the generally known safety profile of sertraline.

**Trial registration:**

ClinicalTrials.gov (Identification No. NCT01607593). Registered May 21, 2012.

## Background

Posttraumatic stress disorder (PTSD) is a psychiatric disorder caused by various traumatic events (e.g., disasters, violence, sexual violence, severe accidents, battles, and child abuse). PTSD is associated with significant symptom-related stress and functional impairment. PTSD shows a high degree of co-morbidity of other anxiety, depression, substance use disorders and suicide [1–3]. Diagnostic and Statistical Manual of Mental Disorders, Fourth Edition (DSM-IV) requires three different types of symptoms to diagnose PTSD; re-experiencing symptoms, avoidance and numbing symptoms, and arousal symptoms [4]. DSM-5 has been released in 2013 and the three clusters of symptoms required in DSM-IV are divided into four clusters; intrusion, avoidance, negative alterations in cognitions and mood, and alterations in arousal and reactivity [5].

Established treatments for PTSD include pharmacological treatments such as antidepressants, and specialized psychologic treatments such as cognitive behavior therapy (CBT) and eye movement desensitization and reprocessing (EMDR) [6]. Pharmacological therapy, though effect size for psychological therapy is reported much larger, has been shown its benefit. Several evidences also show that combining pharmacological treatment and CBT enhances the efficacy of either treatment alone [7]. Most treatment guidelines recognaize certain benefit of pharmacological approach and recommend the use of SSRIs as the first-line drug for PTSD based on evidence from randomized placebo-controlled trials [8]. Pharmacological treatment also has its advantages in patient’s access. It is widely conducted as it can be provided not only by specialists but also by general practitioners, whereas only a limited number of professionals can conduct specialized psychological treatments. Thus, it is expected that pharmacotherapy is practical and convenient, especially in case of the number of PTSD patients may increase suddenly and locally, such as sudden huge disasters.

Sertraline and paroxetine are currently only approved drugs for the treatment of PTSD in the United States and EU countries based on double-blined placebo-controlled randomized clinical trials. These trials demonstrated superiority of the drug to placebo in change from baseline in Clinician Administrated PTSD scale for DSM-IV (CAPS-2) total score [9–13]. To the best of our knowledge, sertraline is the only approved drug that have been demonstrated relapse-prevention effect for PTSD in double-blind placebo-conrolled study [14].

In Japan, sertraline and paroxetine were first approved for the treatment of depression and some anxiety disorders, but not for PTSD, at the time of study initiation. Despite this situation, several studies [15–17] and a multicenter retrospective survey [18] revealed that SSRIs, including sertraline, were used for the treatment of PTSD in Japanese patients. A study from Korea [19], a neighboring country, also showed the effectiveness of sertraline among their veterans. However, there were no published studies investigating actual prescription of sertraline, and its effectiveness and safety for PTSD caused by various types of trauma in Japan. According to the studies in Japan and South Korea, there seem to be not large differences on the efficacy and safety profile of sertraline in patients with PTSD between western and eastern countries. However, because the approved dose range in Japan (25–100 mg/day) is lower than those in other countries (e.g. 50–200 mg/day in the US), it is necessary to examine the efficacy and safety of sertraline in Japanese patients with PTSD separately. This retrospective study was conducted to investigate the dosage regimens, effectiveness, and safety of sertraline for the treatment of PTSD in Japanese real-world clinical settings.

## Methods

### Study design

Following approvals from the institutional ethical review boards of Tokyo Women’s Medical University, Kurume University, Hyogo Institute for Traumatic Stress and National Defense Medical College, we conducted a retrospective chart review of patients who had been treated with sertraline as part of their regular clinical practice, from August to October 2012, at four medical institutions that provide specialized care for PTSD patients in Japan. We investigated the dosage, effectiveness, and safety of sertraline in the treatment of PTSD. This study is presented in part in the clinical trial registry located at ClinicalTrials.gov (Identification No. NCT01607593).

### Study population selection

The study was conducted in the following four medical institutions in Japan; Institute of Women’s Health, Tokyo Women’s Medical University; Department of Neuropsychiatry, Kurume University; Hyogo Institute for Traumatic Stress; and Department of Psychiatry, National Defense Medical College Hospital. Inclusion criteria included (1) male and female outpatients aged 18 years or older; (2) patients who had been diagnosed PTSD by investigators using diagnosis criteria from DSM-IV before the investigators initiated to prescribe sertraline; and (3) patients who had started treatment with sertraline between July 2006 (the start of sertraline marketing in Japan) and October 2012 (the initiation of data entry at the institutions). Exclusion criteria were not set for this study.

### Assessments

All assessments on each subject were performed by the investigator who had diagnosed the patient. Patient characteristics (e.g., age, gender, date of PTSD diagnosis, traumatic events, and medical history), duration and dosage of treatment, treatment status with sertraline (i.e., “completed,” “discontinued,” “continuing”), and concomitant treatments including augumentation treatments (antipsychotics and psychotherapies added to study treatment) were recorded in the Case Report Form. To assess the severity and improvement of PTSD symptoms, the Clinical Global Impressions – Severity (CGI-S) and Clinical Global Impressions – Improvement (CGI-I) ratings [20] were retrospectively determined by investigators based on the medical records. CGI-S was rated at the initiation of sertraline treatment (baseline) and at the end of treatment or the last observation; 1: normal, not at all ill, 2: borderline, mentally ill, 3: mildly ill, 4; moderatery ill, 5: markedly ill, 6: severery ill, 7: among the most extremely ill. CGI-I was rated at the end of treatment or the last observation compared to baseline; 1: very much improved, 2: much improved, 3: minimally improbed, 4: no change, 5: minimally worse, 6: much worse, 7: very much worse. Factors that could influence the effectiveness (e.g., treatment with sertraline for less than 4 weeks, poor medication compliance, and life events such as court litigation) were recorded. All adverse events in the medical charts that were reported during the treatment with sertraline were also recorded. The severity and causal relationship of adverse events with sertraline were determined by the investigators. If the investigator’s final determination of causality is unknown and the investigator does not know whether sertraline caused the event, then the event were handled as related to sertraline for reporting purposes. If the investigator’s causality assessment is unknown but not related to sertraline this should be clearly documented in the Case Report Form. Discontinuation syndrome, self-injury or violence towards others and activation syndrome, are pre-defined as events concerned during treatment SSRIs and focused of this investigation.

### Statistical analyses

The CGI-I and CGI-S ratings were summarized for the full analysis set (FAS), which was defined as all patients with available scores for either CGI-I or CGI-S. Patients who were rated as “very much improved” or “much improved” in the CGI-I rating were defined as responders, whereas the remaining patients were regarded as non-responders. The proportion of responders was referred to as the response rate. The CGI-I ratings were tabulated, and the CGI-S ratings were summarized by cross tabulation of baseline and endpoint data. These analyses of effectiveness were also performed for a subgroup of patients that excluded those with factors that may potentially influence effectiveness. Adverse events were summarized in the safety analysis set, which was defined as all patients included in this chart review. Adverse events were coded by the Medical Dictionary for Regulatory Activities (MedDRA, version 15.1).

To explore patient characteristics that may influence the treatment of PTSD, subgroup analyses of dependent variables (the CGI-I rating, augmentation of treatment and dosage of sertraline) were performed on independent variables defined prior to initiation of data collection: age, duration of disease, concurrent psychiatric disease, traumatic events, and baseline CGI-S. Logistic regression analyses were also performed as pre-defined with responder (vs. non-responder) or at least one treatment augmentation (vs. no treatment augmentation) as the depedent variable, and age, duration of disease, concurrent psychiatric diseases at baseline, traumatic events, and baseline CGI-S as independent variables.

## Results

### Patient characteristics

A total of 122 patients were included in the study. Among the 122 patients, 119 (approximately 98%) were females and three were males (Table 1). The mean age was 35.6 years, and approximately 80% of the patients were under the age of 45. The median duration of PTSD was 2.0 months, ranging from −3.9 months to 74.3 months (the minimum value is a negative figure, because sertraline was prescribed to one patient with suspected PTSD, and the diagnosis was subsequently confirmed). Traumatic events leading to PTSD were physical assault (74 patients), sexual assault (43 patients), child abuse (14 patients), witnessing violence or death (eight patients), captivity (six patients), motor vehicle accident, other accident (five patients each), natural disaster (two patients), fire (one patient) and other (six patients). Ninety-three patients (76.2%) had co-morbid psychiatric disorders at baseline, the most common being depression (53.3%) and dysthymic disorder (8.2%).

**Table 1:** Demographic and clinical characteristics

Characteristics	Sertraline *N* = 122
Age (year), mean (SD)	35.6 (10.4)
Gender distribution	
Male, n (%)	3 (2.5)
Female, n (%)	119 (97.5)
Duration of PTSD (month), mean (range)	2.0 (−3.9 to 74.3)
Traumatic event leading to PTSD, n	
Physical assault	74
Sexual assault	43
Child abuse	14
Witnessing violence or death	8
Captivity	6
Motor vehicle accident	5
Other accident	5
Natural disaster	2
Fire	1
Other	6
Patient with co-morbid psychiatric diseases at baseline, n (%)	93 (76.2)

The median duration of observation was 10.8 months (range 0 – 63 months). Of the 122 patients, 60 (49.2%) discontinued the treatment. The main reasons for discontinuation were insufficient clinical response (16 patients, 13.1%) and adverse events considered related to sertraline (13 patients, 10.7%); another 25 patients (20.5%) were lost to follow-up.

The median duration of treatment with sertraline was 10.6 months (range 0 – 63 months). The dose ranged from 12.5 to 150 mg/day, and the most frequently used doses were 25 and 50 mg/day (43 patients [35.2%] and 35 patients [28.7%], respectively). Only two patients received doses exceeding 100 mg/day (125 and 150 mg/day in one patient each). Concomitant drug and non-drug treatments were used in 121 patients (99.2%) and 16 patients (13.1%), respectively.

### Effectiveness

All 122 patients were included in the FAS. The CGI-I ratings were “very much improved” in 23 patients (18.9%), “much improved” in 38 patients (31.1%), “minimally improved” in 25 patients (20.5%), “no change” in 32 patients (26.2%), and “minimally worse” in four patients (3.3%). The response rate was 50.0% (61 responders). In the subgroup of patients excluding those with factors potentially influencing effectiveness (60 patients), the response rate was 60.0% (36 responders), and only one patient was rated as “minimally worse.”

Table 2 shows a shift table of the CGI-S ratings from baseline to the end of treatment or the last observation (bold face indicated the number of patients who showed improvement in severity from baseline). The CGI-S ratings at baseline were “mild” in 16 patients (13.1%), “moderate” in 68 patients (55.7%), “marked” in 17 patients (13.9%), “severe” in 20 patients (16.4%), and “most severe” in one patient (0.8%). The CGI-S rating at the end of treatment or the last observation showed an overall improvement from baseline, with improvement in severity observed in 80 patients (65.6%), no change in 40 patients (32.8%), and worsening in two patients (1.6%).

In the subgroup of patients that excluded those with factors potentially influencing effectiveness, improvement in severity similar to that seen in the FAS were observed, and there were no patients who exhibited worsening of severity from the baseline level.

**Table 2:** Clinical Grobal Impressions-Severity at baseline and the end of treatment

Baseline *N* = 122		The end of sertraline treatment or the last observation *N* = 122							
		Normal	Borderline	Mild	Moderate	Marked	Severe	Most severe	Not assessed
		13 (10.7)	19 (15.6)	40 (32.8)	40 (32.8)	4 (3.3)	5 (4.1)	1 (0.8)	0
Normal	0	0	0	0	0	0	0	0	0
Borderline	0	**0**	0	0	0	0	0	0	0
Mild	16 (13.1)	**3 (2.5)**	**4 (3.3)**	7 (5.7)	2 (1.6)	0	0	0	0
Moderate	68 (55.7)	**6 (4.9)**	**11 (9.0)**	**26 (21.3)**	25 (20.5)	0	0	0	0
Marked	17 (13.9)	**2 (1.6)**	**1 (0.8)**	**4 (3.3)**	**8 (6.6)**	2 (1.6)	0	0	0
Severe	20 (16.4)	**2 (1.6)**	**3 (2.5)**	**3 (2.5)**	**5 (4.1)**	**2 (1.6)**	5 (4.1)	0	0
Most severe	1 (0.8)	**0**	**0**	**0**	**0**	**0**	**0**	1 (0.8)	0
Not assessed	0	**0**	**0**	**0**	**0**	**0**	**0**	**0**	0

Values represent the number (%) of patients

Bold = patients who showed improvement in severity from baseline

*Normal*, normal, not at all ill, *Borderline* borderline mentally ill, *Mild* mildly ill, *Moderate* moderately ill, *Marked* markedly ill, *Severe* severely ill, *Most severe* most severely ill

### Safety

A total of 73 patients (59.8%) experienced a total of 187 adverse events regardless of causality, and 54 (44.3%) experienced a total of 100 adverse events considered to be related to sertraline. Table 3 represents the adverse events observed in five or more patients. The most commonly observed adverse events (≥10 patients) were nausea and headache (12 patients, 9.8%, each) and somnolence (11 patients, 9.0%), most of which were considered to be treatment-related. Sixteen patients (13.1%) discontinued sertraline and eight patients (6.6%) received a reduced dose or temporarily suspended sertraline due to adverse events, most of which were considered treatment-related. Discontinuation syndrome, self-injury or violence towards others, and activation syndrome, which are events of concern during treatment with SSRIs, were observed in one patient (0.8%), nine patients (7.4%), and one patient (0.8%), respectively. The treatment-related adverse events that caused discontinuation or dose reduction in multiple patients were nausea (five patients), somnolence (three patients), as well as headache and dry mouth (two patients each). Seven patients (5.7%) reported serious adverse events (acute myeloid leukaemia; pituitary tumour; suicide attempt; intentional overdose; intentional self-injury: ovarian neoplasm; hallucination, thinking abnormal); and two patients (1.6%) experienced severe adverse events (ovarian neoplasm and acute myeloid leukaemia), but none of these serious or severe adverse events were considered to be related to sertraline.

**Table 3:** Adverse events reported in ≥ 5 patients regardless of causality

MedDRA version 15.1 Preferred term	All-Causality (*N* = 122)	Treatment-Related (*N* = 122)
Abdominal discomfort	5 (4.1)	3 (2.5)
Abdominal pain upper	5 (4.1)	2 (1.6)
Diarrhoea	7 (5.7)	4 (3.3)
Dry mouth	7 (5.7)	6 (4.9)
Nausea	12 (9.8)	11 (9.0)
Intentional overdose	5 (4.1)	0
Headache	12 (9.8)	8 (6.6)
Somnolence	11 (9.0)	10 (8.2)

Values represent the number (%) of patients

### Investigation of factors potentially influencing the treatment outcome of PTSD

**Fig. 1:**
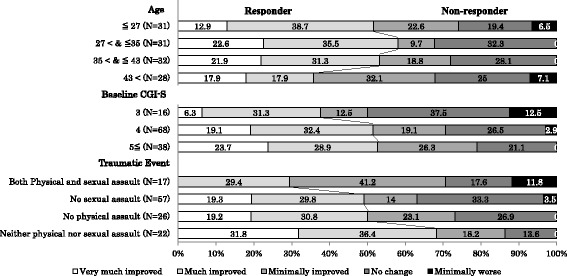
Propotion of responder in Clinical Grobal Impression-Improvement Ratings by patient characteristics. Responders: patients who were rated as “very much improved” or “much improved” in the CGI-I rating. Values in the bar chart represent the proportion (%) of patients. Age-subgroups were divided by the quartiles. No sexual assault: patients experienced traumatic events including physical assault, but no sexual assault. No physical assault: patients experienced traumatic events including sexual assault, but no physical assault

Subgroup analyses of the CGI-I ratings by patient characteristics suggested that PTSD symptoms improved to a lesser degree in patients older than 43 years, those with mild severity at baseline (CGI-S score of 3), and those who had experienced both physical and sexual assault as trauma (Fig. 1). Logistic regression analyses indicated that the type of traumatic events was the factor with the highest influence on the response rate among the explanatory variables we selected (Table 4). In particular, the response rate in patients who had experienced both physical and sexual assault was much lower than in those who experienced neither (odds ratio, 0.12; 95% confidence interval, 0.025 to 0.556).

**Table 4:** Logistic regression analysis: responder vs. non-responder

Factor	d.f.	Chi-Square statistic	*p*-value	Odds ratio estimates			
					Point estimate	95% C.I.	
Age	1	1.75	0.186		0.97	0.94	1.01
Duration of disease	1	0.26	0.610		0.99	0.97	1.02
Baseline CGI-S	1	0.43	0.510		1.15	0.76	1.76
Concurrent psychiatric disease at baseline	2	1.61	0.447	Depression or Dysthymic disorder vs. None	1.74	0.64	4.74
				Other Psychiatric disease vs. None	2.23	0.58	8.55
Traumatic events	3	7.43	0.060	Both Physical and sexual assault vs. Neither	0.12	0.03	0.56
				No sexual assault vs. Neither	0.38	0.12	1.20
				No physical assault vs. Neither	0.30	0.08	1.12

*d.f*. Degree of freedom, *C.I.* Confidence interval

Responders: patients who were rated as “very much improved” or “much improved” in the CGI-I rating

No sexual assault: patients experienced traumatic events including physical assault, but no sexual assault

No physical assault: patients experienced traumatic events including sexual assault, but no physical assault

**Fig. 2:**
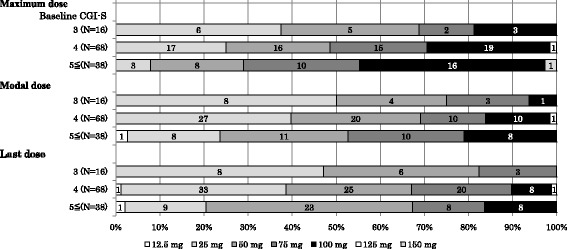
Dosage (mg/day) by baseline CGI-S score. Values in the bar chart represent the number of patients

**Fig. 3:**
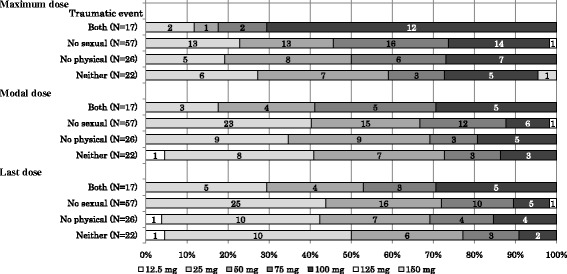
Dosage (mg/day) by traumatic event. Values in the bar chart represent the number of patients. Both: patients experienced traumatic events both physical assault and sexual assault. No sexual: patients experienced traumatic events including physical assault, but no sexual assault. No physical: patients experienced traumatic events including sexual assault, but no physical assault. Neither: patients experienced traumatic events neither physical assault or sexual assault

Subgroup analyses also suggested that augmentation of treatment (prolonged exposure therapy, antipsychotics or mood stabilizer) was more likely in patients who had experienced sexual assault as trauma (Table 5), and that a higher dose was more likely to be administered to patients with severe symptoms at baseline (CGI-S score of ≥5) and those who experienced both physical and sexual assault as trauma (Figs. 2 and 3). Augmentation of treatment was most highly affected by the type of the traumatic events among the five explanatory variables (Table 6). In particular, there was a much higher possibility of augmentation of treatment in patients who experienced traumatic events including sexual assault, but not physical assault than in those who experienced neither (odds ratio, 3.80; 95% confidence interval, 1.026 to 14.056).

**Table 5:** Augmentation of treatment by traumatic event

Traumatic events	Both Physical and sexual assault (*n* = 17)	No sexual assault (*n* = 57)	No physical assault (*n* = 26)	Neither physical nor sexual assault (*n* = 22)
Two treatments	5 (29.4)	7 (12.3)	4 (15.4)	4 (18.2)
One treatment	5 (29.4)	13 (22.8)	16 (61.5)	6 (27.3)
None	7 (41.2)	37 (64.9)	6 (23.1)	12 (54.5)
Treatment type				
PE therapy	2 (11.8)	4 (7.0)	4 (15.4)	2 (9.1)
Antipsychotics	10 (58.8)	17 (29.8)	16 (61.5)	8 (36.4)
Mood stabilizer	3 (17.6)	6 (10.5)	4 (15.4)	4 (18.2)

*PE* prolonged exposure therapy

**Table 6:** Logistic regression analysis: at least one treatment augmentation vs. no treatment augmentation

Factor	d.f.	Chi-Square statistic	*p*-value	Odds ratio estimates			
					Point estimate	95% C.I.	
Age	1	0.40	0.526		1.01	0.97	1.05
Duration of disease	1	0.04	0.836		1.00	0.98	1.03
Baseline CGI-S	1	0.22	0.638		1.11	0.73	1.69
Concurrent psychiatric disease at baseline	2	0.46	0.793	Depression or Dysthymic disorder vs. None	1.35	0.49	3.76
				Other Psychiatric disease vs. None	1.54	0.40	5.94
Traumatic events	3	11.54	0.009	Both Physical and sexual assault vs. Neither	1.43	0.35	5.75
				No sexual assault vs. Neither	0.57	0.19	1.64
				No physical assault vs. Neither	3.80	1.03	14.06

*d.f.* Degree of freedom, *C.I.* Confidence interval

No sexual assault: patients experienced traumatic events including physical assault, but no sexual assault

No physical assault: patients experienced traumatic events including sexual assault, but no physical assault

## Discussion

Our study indicated that sertraline was well-tolerated, and the PTSD symptoms, according to the CGI-I rating, improved during sertraline treatment. In Japan, the approved sertraline dose for major depressive disorder and panic disorder was lower dose than that in the United States (25–100 mg/day vs. 50–200 mg/day), and most of the study patients were treated within this dose. Nonetheless, our findings were consistent with previous American findings [9, 10]. Our study data were collected from medical charts of all PTSD patients treated with sertraline who visited the four specialized medical institutions in Japan where they received regular clinical practice. This mminimized the selection bias of patients. The results are also considered to reflect the usage of sertraline in the regular clinical practice and might be extrapolated to similar medical institutions in Japan.

The majority of our study patients were female (97.5%), so there were not enough data to examine gender differences within our study group. An American community study had suggested that females were approximately twice as likely as males to develop PTSD (lifetime prevalence; females, 10.4% vs. males, 5.0%) [21]. A similar trend was observed among the Japanese population (1.3% among females and 0.6% among males) [22]. These studies indicated that females had a higher prevalence of PTSD than males, whereas meta-analysis of pharmacotherapy for PTSD indicated that gender did not affect treatment outcome [8]. There were also no gender-related differences in treatment outcomes among depressed patients who were treated with sertraline [23]. Our study might suggest that PTSD can be treated with sertraline regardless of gender, but further studies will be needed to validate this theory.

Our analysis showed a tendency for lower CGI-I response rate among patients who experienced both physical and sexual assault as their traumatic events. Although 70.6% of patients who experienced both events showed improvement, the response rate in this subgroup was 29.4%, whereas it was 68.2% in patients who experienced neither physical nor sexual assault. Logistic regression analysis also showed a similar trend. In addition, a higher dose of sertraline was observed in a higher portion of patients with physical and sexual traumatic events. A marked difference in the usage of antipsychotic drugs between the different trauma types were shown; patients who experienced sexual assault were more likely to have add-on treatment with antipsychotic drugs regardless of whether they had experienced physical assault compared to those without sexual assault. Note that these findings may be confounded by PTSD severity/chronicity, which may depend on traumatic experiences.

The present study collected type of traumatic events leading to PTSD as background but did not collect data on number of traumatic events experienced. We were thus not able to investigate differences in treatment outcome and predicitive impact between multiple and single events of trauma. However, the observation that patients with physical and sexual trauma are more likely to have treatment-resistant PTSD suggests that the diagnosis and identification of the types of traumatic events are important to determine the treatment strategy. On the other hand, a higher response rate in patients who experienced neither physical nor sexual assault suggests that pharmacological treatment may provide greater benefit to patients with single trauma. A population-based, cross-national chart review suggested that patients with PTSD associated with multiple traumatic events or repeated exposure to the same traumatic event had severer morbidity and impairment compared to those patients with PTSD caused by a single traumatic event [24]. Further research is required to examine the treatment outcome by type or number of traumatic events.

This study was a retrospective study and it includes some limitations. First, the nature and the design of the investigation limited the data collection including assessments by rating scales and there still is a possibility of residual confounding and inherent bias in the data collected. Second, as sertraline dose had been adjusted by the each patient’s symptoms and/or conditions during PTSD treatment, therefore we could not examine dose response relationship of efficacy among Japanese PTSD patient. Sertraline dose is considered as one of the response variables and We are also not able to include the dosing information in the logistic regression model. Third, the number of patients was variable among the four institutions and this may cause imbalance among the institutions in patient characteristics such as gender. Finally, a relatively large percentage of patients discontinued sertraline, although this trend has been seen in other surveys conducted in Japan [18, 25]. Given these limitations, our findings indicated that the symptoms of PTSD improved during treatment with sertraline without additional safety concerns. Further prospective controlled studies are needed to confirm our findings.

## Conclusions

The results of this study suggested the effectiveness of sertraline for the treatment of PTSD, and the obtained safety profile was consistent with the generally known safety profile of sertraline.

## Abbreviations

CAPS-2: Clinician Administrated PTSD scale for DSM-IV
CBT: Cognitive behavior therapy
CGI-I: Clinical Global Impressions – Improvement
CGI-S: Clinical Global Impressions – Severity
DSM: Diagnostic and statistical manual of mental disorders
EMDR: Eye movement desensitization and reprocessing
FAS: Full analysis set
MedDRA: Medical Dictionary for Regulatory Activities
PTSD: Posttraumatic stress disorder (PTSD)
SSRI: Selective Serotonin reuptake inhibitor
